# The Brain-Heart Connection in Takotsubo Syndrome: The Central Nervous System, Sympathetic Nervous System, and Catecholamine Overload

**DOI:** 10.1155/2020/4150291

**Published:** 2020-03-09

**Authors:** Xiaopu Wang, Junyu Pei, Xinqun Hu

**Affiliations:** Department of Cardiovascular Medicine, The Second Xiangya Hospital, Central South University, Changsha, 139 Middle Renmin Road, Hu'nan 410011, China

## Abstract

Takotsubo syndrome (TTS), also known as stress cardiomyopathy, is a type of acute heart failure syndrome triggered by intense psychological or physiological stress. TTS typically manifests as acute chest pain, dyspnea or syncope that mimics an acute myocardial infarction but does not involve coronary artery obstruction. The current understanding of the pathogenesis of TTS suggests that sympathetic nervous system (SNS) activation plays a central role. Specifically, stress can activate the SNS and lead to the over-release of catecholamine, which have toxic effects on myocardial tissue when present at excessive levels. However, the brain changes associated with TTS and the connection between the brain and the heart in patients with this disease remain unclear. In recent years, several published reports have revealed the role of this brain-heart connection in the pathogenesis of TTS. This review summarizes recent studies regarding SNS activation, catecholamine overload, and the brain-heart connection in patients with TTS from both pathophysiological and mechanistic aspects.

## 1. Introduction

Takotsubo syndrome (TTS), which is also known as stress cardiomyopathy, is an acute syndrome that is induced by psychological or physiological stress and characterized by acute reversible heart failure [1]. TTS typically manifests as the acute onset of chest pain, dyspnea, or syncope, and may even present as ventricular arrhythmia or cardiogenic shock in severe cases [2]. Initially, it may be difficult to distinguish TTS from acute myocardial infarction (AMI), as the electrocardiogram (ECG) of a patient with TTS would display an ST-segment elevation with or without an elevated troponin or creatine kinase-MB (CK-MB) level [3]. However, most patients with TTS do not present with coronary artery occlusion, and such cases are also characterized by reversible left ventricular dysfunction [3].

TTS was first described by Sato et al. in a report of five cases [4]. In that series, the first case involved a 64-year old woman with significant symptoms and ECG changes consistent with AMI. However, her coronary arteries were normal, and apical ballooning gave the appearance of a traditional Japanese pot called a “Takotsubo.” She additionally presented with marked abnormalities in ventricular motion that were visible on left ventriculography, which disappeared after 2 weeks [5]. These interesting cases were first reported only in Asia, and the disease attracted gradual attention as western countries began to report similar cases in the late 1990s [6]. In 2006, the American Heart Association (AHA) officially conferred the name “stress cardiomyopathy” on this condition [7]. In 2015, however, the European Society of Cardiology (ESC) proposed to abandon “cardiomyopathy” in favor of the original term, takotsubo syndrome (TTS), in light of recent basic and clinical research [1].

As noted above, the number of reported cases of TTS worldwide increased gradually. Current epidemiological data indicate that TTS patients account for 1–3% of all cases of suspected AMI, and 90% of affected patients are postmenopausal women [8–10], consistent with the observation that women older than 55 years have a 10-fold greater risk of TTS than men of the same age and a five-fold greater risk than younger women [11]. In the United States, TTS accounts for 0.02% of hospitalizations and has an in-hospital mortality rate of approximately 2% [11, 12]. Despite some severe and potentially fatal complications, including heart failure, cardiac shock, and malignant arrhythmias, the prognosis of TTS is generally favorable [13].

## 2. The Role of Stress and Catecholamines in Takotsubo Syndrome

Although the pathophysiological mechanism of TTS is incompletely understood, the syndrome is generally considered a complex and systemic cardiovascular system reaction caused by acute and severe psychological or physiological stimulation [1]. Stress events are considered both a hallmark of TTS and the most important general trigger. The results of the Comorbidity Frequency in the Takotsubo Syndrome (COUNTS) study indicated that emotional and physical stress were the triggers in 39% and 35%, respectively, of 1109 patients with TTS and that 24% of affected patients had mental disorders [14]. Moreover, Summers et al. reported in 2010 that many women with TTS had a history of chronic anxiety before onset [15]. In patients, physical stress may be related to basic disease. For example, the COUNTS study found as many as 15% of patients with TTS had pulmonary vascular disease, while 7% had nervous system disease (subarachnoid hemorrhage was most common) and 1% had experienced trauma [14]. Another study identified complications such as malignant tumors, chronic kidney diseases, and connective tissue diseases as the strongest predictors of death in patients with TTS [16].

In the current concept of TTS, increasing sympathetic nervous system (SNS) activity plays a central part in the disease pathogenesis. A stress event triggers SNS activation, leading to the release of catecholamines. A recognizable emotionally or physiologically triggering event and excess catecholamines release have been identified in most cases of TTS [17–20]. In 2005, Wittstein et al. observed significantly higher levels of catecholamines in patients with TTS than in patients with Killip class III myocardial infarction [21]. However, this finding has not been duplicated by other studies, possibly because of limitations of the methodology or number of cases. In a 2009 study, Madhavan et al. did not identify elevated plasma catecholamines concentrations but did detect an interesting marked increase in plasma noradrenaline concentrations at the onset of TTS in a cohort of 15 patients [22]. In 2008, Kume et al. observed elevated noradrenaline concentrations in the coronary sinuses of patients with TTS [23]. Although these studies suggest an association between SNS activation and TTS, the plasma catecholamines concentration is not necessarily related to local myocardial sympathetic regulation [24].

Despite the abovementioned conflicting results, TTS has been induced in several clinical cases by the intravenous injection of catecholamines. In 2017, Kido and Guglin analyzed 157 cases of drug-induced TTS and found that 68.2% were catecholamines-related, while 8.9% appeared to be associated with chemotherapy-induced coronary vasospasm [25]. In addition, Abraham et al. revealed in 2009 that the intravenous administration of epinephrine or beta-receptor agonists could induce all the characteristic features of TTS, including an elevation of cardiac isoenzyme levels, rapidly reversible cardiac dysfunction, and QTc interval prolongation [26]. Notably, some studies of pheochromocytoma and other diseases associated with the excess release of catecholamines have provided evidence supporting the abovementioned observations. For example, Giavarini et al. observed 140 consecutive patients with pheochromocytomas and paragangliomas (PPGL) and considered that the latter condition may present as acute catecholamine cardiomyopathy (ACC) in 11% of cases (excluding patients who died from undiagnosed tumors) [27, 28]. Several studies of animal models of TTS have led to similar conclusions [29]. Immobilization stress can provoke apical ballooning of the left ventricle in rats, while alpha- and beta-receptor blockade can attenuate this ballooning [30, 31].

Mechanistically, the transient left ventricle dysfunction observed in TSS may represent the toxic effect of an excess of catecholamines on the myocardium. At doses above normal physiological levels, catecholamines can disrupt the calcium-regulatory system by stimulating *β*-adrenoceptors and consequently downregulating the expression of genes encoding calcium-regulatory protein [32]. Sarcoplasmic-Ca^2+^-ATPase (SERCA2a) gene expression is downregulated with the upregulation of sarcolipin, while phospholamban is dephosphorylated. The consequent increase in the phospholamban/SERCA2a ratio leads to contractile dysfunction via decreased affinity for Ca^2+^ [33]. In patients with acute-phase TTS, the histopathological features of this contractile dysfunction include regional inflammatory cell infiltration, enhanced fibrosis, and contraction bands [34].

## 3. Activation of the Sympathetic Nervous System (SNS) in Takotsubo Syndrome

Other aspects of SNS activation have also been observed in patients with TTS. As mentioned above, 90% of patients with TTS are postmenopausal women. During this unique period of life, a decrease in estrogen levels weakens the parasympathetic nerve stabilization in the hypothalamic autonomic center, which increases the reactivity of the SNS to activation. We believe that this increased reactivity may at least partly explain why TTS most frequently affects postmenopausal women. Interestingly, a study of 33894 patients with TTS (88.9% women) found that the prevalence of diabetes mellitus was lower than the prevalence in a general population of participants in the National Health and Nutrition Examination Survey (NHANES) [35]. We hypothesize that the autonomic neuropathy induced by diabetes mellitus results in a disconnection between the brain and heart disconnection and thus could conceivably alleviate the characteristic effect of an adrenergic storm of the myocardium in TTS.

As noted above, the ECG characteristics observed in TTS patients are very similar to those in AMI patients. In both conditions, the most common manifestations are T-wave inversion, large upright peaked T-waves and QT interval prolongation. Possibly, the ECG changes observed in TTS may reflect the disturbance of the sympathetic nerve terminals [36]. In a review of the 24-hour ambulatory electrocardiograms of TTS patients with apical ballooning at 2 and 3 days and 3 months, Ortak et al. observed that the indices of heart rate variability (HRV) were significantly depressed, suggesting a decrease in cardiac parasympathetic activity [37]. Akashi et al. reached a similar conclusion when exploring the standard deviation of the mean cycle lengths of normal-normal R-R (NN) intervals over 24 h (SDNN). In that study, the 24-h standard deviation of the mean value of the difference between NN intervals for each 5-min segment (SDANN) had improved significantly at 3 months post-onset in patients with TTS [38]. Nuclear imaging may provide more evidence supporting a role of the SNS in TTS. For example, an earlier study revealed that a decrease in ^123^I-metaiodobenzylguanidine uptake on single-photon-emission computed tomography was indicative of cardiac sympathetic hyperactivity in patients with TTS [39].

## 4. Brain-Heart Connection in Takotsubo Syndrome

Stress is a physiological response mediated by both the central nervous system and SNS. The limbic system, neocortex, spinal cord, reticular formation, and brainstem are fundamental anatomic structures in the stress response [40]. In this response, the main neuroendocrine changes that occur in response to strong stimulation include an intense excitation of the locus coeruleus-adrenomedullin axis and hypothalamus-pituitary-adrenocortical (HPA) axis [41].

The locus coeruleus is situated in the posterior area of the rostral pons in the lateral floor of the fourth ventricle and serves as the central site of noradrenergic neurons in the brain stem and sympathetic adrenomedullin system. The locus coeruleus can receive afferent signals from the amygdala, hypothalamus, and cingulate gyrus and is related to excitement and alertness during stress. Moreover, this brain structure can cause emotional reactions such as tension and anxiety, which can trigger noradrenergic responses. The locus coeruleus also regulates the acute response of the body to stress by maintaining a state of alertness that is conducive to coping with environmental changes. Activation of the locus coeruleus induces the secretion of norepinephrine by adrenal medullary chromaffin cells, which in turn stimulates the HPA axis [42, 43].

The sympathetic nerve descends through the cranial and sacral spinal cord. Sympathetic preganglionic neurons are located in the lateral gray column between the spinal levels of T1 and L2. These neurons form synapses with postganglionic neurons and then interact with the myocardium and coronary circulation along the epicardial vessels. The sympathetic nerve endings activate the *α* and *β* postsynaptic adrenergic receptors by releasing noradrenaline into the synaptic space [44].

Stress is also regulated by the HPA axis, which is also known as the limbic system-hypothalamus-pituitary-adrenal axis (LHPA axis). This axis is centered around the paraventricular nucleus, which comprises the hypothalamus, adenohypophysis, and adrenal cortex. Notably, cortisol synthesis in the adrenal cortex is the downstream result of HPA axis activity. Cortisol, a major stress hormone, can act on many tissues, including the brain, and the combined functions of the sympathetic nerve and cortisol can promote the synthesis and secretion of adrenaline and noradrenaline in the adrenal medulla. The hypothalamus is connected physically to the amygdala and hippocampus, among other structures, and these nuclei can also stimulate the HPA axis via these physical links.

Recently, increasing interest has been directed toward the brain and neural changes observed in patients with TTS. In 2014, Suzuki et al. used (99 m)Tc ethyl cysteinate dimmer single-photon-emission computed tomography to measure the cerebral blood flow (CBF), a widely accepted index of brain activity, in patients during the acute and chronic phases of TTS. In the acute phase, the researchers observed a marked increase in CBF in the brainstem, hippocampus, and basal ganglia, which was accompanied by a significant decrease in CBF in the prefrontal cortex [45]. Both the basal ganglia and hippocampus are components of the limbic system, which is associated with emotion and sympathetic activation, while the brainstem contains the sympathetic central nucleus and the origins of descending sympathetic nerves. In 2017, Klein et al. similarly demonstrated specific homogeneous anatomical and neurophysiological features in brain regions mainly associated with the control of heart functions in patients with TTS [46]. In 2018, Hiestand et al. used magnetic resonance imaging to reveal structural and connective differences in the limbic networks of TTS patients and healthy subjects. Specifically, patients with TTS had a cortex over the limbic region and significantly reduced connectivity in the autonomic nervous system, including the left amygdala, both hippocampi, the left superior temporal pole, and right putamen [47]. Moreover, a recently published study similarly reported reduced functional connectivity in the limbic systems of patients with TTS patients relative to healthy individuals [48]. As noted above, the proportion of individuals with mental disorders was found to be higher among patients with TTS than in a general population. Therefore, primary structural alterations in the autonomic nervous systems of TTS patients may reduce control in this region.

## 5. Conclusions and Perspectives

**Figure 1:**
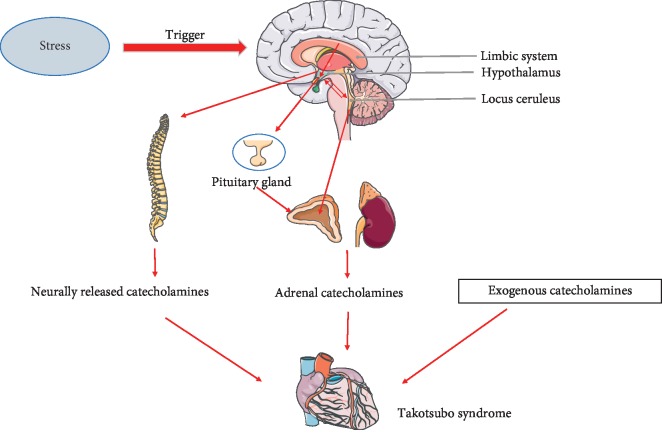
The possible brain-heart connection in the pathogenesis of TTS. The stress-induced alterations in the central nervous system may activate the SNS and thus cause TTS.

In this review, we have presented the findings of recent studies concerning the potential pathophysiologic and mechanistic roles of SNS activation, catecholamine overload, and the brain-heart connection in patients with TTS. The mechanisms by which an excess of catecholamines induce direct and indirect myocardial damage have been clarified gradually over time, and the role of the SNS in the pathogenesis of TTS has become clear. Notably, several recently published reports revealed that stress-related structures in the brain undergo anatomical and neurophysiological changes during the onset of TTS, suggesting that stress-induced alterations in the central nervous system may activate the SNS and thus cause TTS (Figure 1). However, these studies all featured a cross-sectional design. Additional randomized prospective trials and new interdisciplinary approaches are required to further investigate the role of the central nervous system and the brain-heart connection in the pathogenesis of TTS.
